# Supplementary material for: Diagnostic accuracy of the Copenhagen Index in ovarian malignancy: A meta-analysis
Source: PLoS One. 2023 Jun 14;18(6):e0286650. doi: 10.1371/journal.pone.0286650 (PMC10266642; doi:10.1371/journal.pone.0286650)
Supplement: Attachment — Submitted filename: Respond to Editor and Reviewers.docx [file pone.0286650.s003.docx]

**Dear editor and reviewers,**

**Re: “Diagnostic accuracy of the Copenhagen Index in ovarian malignancy: a meta-analysis " (PONE-D-21-26313)**

Thanks to the editor and reviewers for the insightful comments and the valuable suggestions for revision of our MS. We have made changes in accordance with the suggestions from the editor and the reviewers in our revised MS. All of the modifications are highlighted in red in the text. The point by point response to the editor and reviewers’ comments was attached in the following part. We hope the changes incorporated would make the manuscript acceptable for publication in the Journal of PLOS ONE.

**Editor Comments:**

**Question 1:** Please ensure that your manuscript meets PLOS ONE's style requirements, including those for file naming.

**Response:** Thanks for your question. We have examined the MS and ensured that our manuscript meets PLOS ONE's style requirements.

**Question 2 :** Thank you for providing the date(s) when patient medical information was initially recorded. Please also include the date(s) on which your research team accessed the databases/records to obtain the retrospective data used in your study.

**Response:** Thanks for your question. We have provided the date on which our research team accessed the databases/records to obtain the retrospective data used in our study in Search strategy section.

**Question 3 :** We note that the grant information you provided in the ‘Funding Information’ and ‘Financial Disclosure’ sections do not match. When you resubmit, please ensure that you provide the correct grant numbers for the awards you received for your study in the ‘Funding Information’ section.

**Response:** Thanks for your question. We have provided the correct grant numbers for the awards.

4. In your Data Availability statement, you have not specified where the minimal data set underlying the results described in your manuscript can be found. PLOS defines a study's minimal data set as the underlying data used to reach the conclusions drawn in the manuscript and any additional data required to replicate the reported study findings in their entirety. All PLOS journals require that the minimal data set be made fully available. For more information about our data policy, please see http://journals.plos.org/plosone/s/data-availability.

**Response:** Thanks for your question. The appropriate data supporting the conclusions of this article is included within the Supporting information:S1 file.

**Reviewer 1:**

**Question 1 :** Line 70: During the last 5 years, the international literature on Copenhagen Index (CPH-I) has been increased. Authors are recommended to recheck PubMed for papers (up to June 2021, as stated) for further eligible studies, i.e. check the article PMID: 33994145 for eligibility for inclusion.

**Response:** Thanks for your question insightful comment. We have checked the article PMID: 33994145 for eligibility for inclusion, which did not meet our inclusion criteria.

**Question 2:** Lines 197-199: the authors are recommended to provide more discussion on the value of CPH-I in non-epithelial ovarian /adnexal tumors
**Response:** Thanks for your question. We have provided more discussion on the value of CPH-I in non-epithelial ovarian /adnexal tumors.

**Question 3:** Lines 210-211: It’s recommended to place the limitations of the study at the end of Discussions, not within the Conclusion.

**Response:** Thanks for your question. We have placed the limitations of the study at the end of Discussions.

**Question 4:** • Line 215: “…because small studies with null results tend to remain unpublished,…”: this comment/judgement is too subjective and should be re-formulated.
• Check English syntax and grammar

**Response:** Thanks for your question. We have modified the English syntax and grammar: The potential for publication bias is present in this article, as small studies with null results are often unpublished, and there may also be bias in outcome reporting.

**Reviewer 2:**

This manuscript of Liu et colleagues (entitled Diagnostic accuracy of the Copenhagen Index in ovarian malignancy: a meta-analysis) addresses an interesting topic. The Copenhagen Index algorithm (CPH-I), published by Karlsen et al in 2015, considered three elements: measurements of CA125, HE4, and patient’s age. The serum CA125 is strictly related to the stage of the disease: higher serum levels are present in 80%–90% of women with stage III–IV disease, but approximately in 50% of stage I disease. HE4 has higher specificity and lower false-positivity than CA125, but could be associated with others disease (as lung and endometrial cancer, renal disease or endometriosis). In comparison to ROMA score, CPH-I doesn’t consider the menopausal status but only the age of the patient. In fact, menopause diagnosis is not standardized, thus HE4 serum levels increase with age regardless of menopause. CPH-I was as effective as ROMA and superior to RMI. The gold standard of different diagnosis is considered gynecological imaging like transvaginal ultrasound. However this exam depends on skill level of the operator. Thus, in non-specialized centers auxiliary methods such as CPH-I and ROMA may be useful to refer selected patients with malignant disease to a specialized cancer center. I appreciate the novelty of the paper, and the design of the study; all important data are reported and figures are appropriate and useful. Method section is complete. Statistical analysis is well conducted and adequate to highlight the potential clinical role and technical reliability . I advice to update the background section with a more complete description of Copenhagen Index to provide a complete overview. In the Results I suggest to introduce a separate “Quality assessment” section. I also suggest to considered this paper DOI: 10.1016/j.ygyno.2021.05.001 about the evaluation of ROMA vs CPH-I, which showed a median values of CPH-I and ROMA lower than those of some other studies. In conclusion, discerning a benign from a malignant tumor before surgery is crucial to the correct referral the woman, considering that survival of ovarian cancer patients is highly dependent on the primary surgical and oncological treatment. In non-specialized centers, triaging methods such as CPH-I and ROMA can be more useful to select patients for referral to highly specialized gynaecological cancer centers. In clinical practice the CPH-I might be considered as an alternative to ROMA, with a similar accuracy but simpler, easy to collect and more objective.

**Response:** Thanks for your question and insightful comment. We have updated the background section. CPH-I, a new tumor index calculated by HE4, CA125, and age rather than menopausal status with different definitions, was thought to be easier to obtain than ROMA clinically. Researches have shown that CPH-I and ROMA have similar discriminatory performance in benign lesions and malignant ovarian tumors

Thank you for your kind consideration.

Sincerely,

Dr. Xiaoling Gao, professor, Director

The Institute of Clinical Research and Translational Medicine

Gansu Provincial Hospital

No. 204 Donggang West Road, Chengguan District, Lanzhou, 730000, China.

E-mail addresses: gaoxl008@hotmail.com

Tel: +86-931-8281222;

Fax: +86-931-8281222.
