# Supplementary material for: Diagnostic accuracy of the Copenhagen Index in ovarian malignancy: A meta-analysis
Source: PLoS One. 2023 Jun 14;18(6):e0286650. doi: 10.1371/journal.pone.0286650 (PMC10266642; doi:10.1371/journal.pone.0286650)
Supplement: Attachment — Submitted filename: Response to Reviewers.docx [file pone.0286650.s004.docx]

**Dear editor and reviewers,**

**Re: “Diagnostic accuracy of the Copenhagen Index in ovarian malignancy: a meta-analysis " (PONE-D-21-26313R1)**

Thanks to the editor and reviewers for the insightful comments and the valuable suggestions for revision of our MS. We have made changes in accordance with the suggestions from the editor and the reviewers in our revised MS. All of the modifications are highlighted in red in the text. The point by point response to the editor and reviewers’ comments was attached in the following part. We hope the changes incorporated would make the manuscript acceptable for publication in the Journal of PLOS ONE.

**Editor Comments:**

**Question 1:** Please review your reference list to ensure that it is complete and correct. If you have cited papers that have been retracted, please include the rationale for doing so in the manuscript text, or remove these references and replace them with relevant current references. Any changes to the reference list should be mentioned in the rebuttal letter that accompanies your revised manuscript. If you need to cite a retracted article, indicate the article’s retracted status in the References list and also include a citation and full reference for the retraction notice.

**Response:** Thanks for your question. We have examined the reference list that it is correct. In addition, we have added a reference [basing on](javascript:;) the comment of Reviewer 3 , which was highlighted in red in the reference list.

**Reviewer 3:**

**Question 1 :**Please, modify "maging" with imaging in 44 line.
I reccomend to add a reference for the sentence: "Carbohydrate antigen 125 (CA125) has recently been recognized as a method for screening and evaluating ovarian cancer, but it is prone to false positives and has low specificity and sensitivity for the detection of ovarian cancer".

**Response:** Thanks for your question.We have modified "maging" with imaging in 44 line. We have added a reference for the sentence: "Carbohydrate antigen 125 (CA125) has recently been recognized as a method for screening and evaluating ovarian cancer, but it is prone to false positives and has low specificity and sensitivity for the detection of ovarian cancer".It was highlighted in red in the reference list.

Thank you for your kind consideration.

Sincerely,

Ms Shouye Ma, MD

Obstetrics and Gynecology Department

Gansu Provincial Hospital

No. 204 Donggang West Road, Chengguan District, Lanzhou, 730000, China.

E-mail addresses: [msymashouye@163.com](mailto:msymashouye@163.com)

Tel: +86-931-8281060;

Fax: +86-931-8281060.
